# Estimating the risk of suicide associated with mental disorders: A systematic review and meta-regression analysis

**DOI:** 10.1016/j.jpsychires.2021.02.053

**Published:** 2021-05

**Authors:** Modhurima Moitra, Damian Santomauro, Louisa Degenhardt, Pamela Y. Collins, Harvey Whiteford, Theo Vos, Alize Ferrari

**Affiliations:** a Institute for Health Metrics and Evaluation, University of Washington, United States; b The University of Queensland, School of Public Health, Queensland, Australia; c Queensland Centre for Mental Health Research, Queensland, Australia; d National Drug and Alcohol Research Center, University of New South Wales, Australia; e Department of Global Health, University of Washington, United States; f Department of Psychiatry and Behavioral Sciences, University of Washington, United States

**Keywords:** Suicide, Mental disorders, Systematic review, Meta-regression, Global mental health

## Abstract

**Background:**

Mental disorders (MDs) are known risk factors for suicide. This systematic review updates the evidence base for this association and improves upon analytic approaches by incorporating study-level and methodological variables to account for measurement error in pooled suicide risk estimates**.**

**Methods:**

A systematic review was conducted to review studies on MDs as risk factors for suicide. Relevant studies were searched using PubMed, Embase, PsychINFO, and existing reviews from 2010 to 19. Studies were eligible if they were longitudinal/case-control studies, representative of the general population, used diagnostic instruments, and quantified suicide risk. The outcome assessed was relative risks (RRs) for suicide due to MDs. A multi-level meta-regression approach was used to obtain pooled RRs adjusted for covariates and between-study effects.

**Findings:**

We identified 20 eligible studies yielding 69 RRs. Disorder type, age, sex, use of psychological autopsy, study design, and adjustment for confounders were tested as predictors of pooled suicide risk. Overall, all disorders were significant predictors of suicide with predicted adjusted RRs ranging from 4·11 [2·09, 8·09] for dysthymia to 7·64 [4·3, 13·58] for major depressive disorder.

**Interpretation:**

Our results indicate that MDs are important risk factors for suicide. This systematic review provides pooled RRs that have been adjusted for methodological sources of bias. Findings from our paper may inform suicide prevention strategies as part of national health agendas.

## Highlights

• Mental disorders are important risk factors for suicide.
• Differences in study methodology may contribute to over or underestimation of study-reported suicide risk.
• It is important to analytically quantify sources of variation when synthesizing published evidence on suicide risk in a meta-regression framework.

## Introduction

The Global Burden of Disease Study (GBD) estimated suicide to be the 15th leading cause of years of life lost (YLLs) in 2019, up from the 18th in 2010 (Vos et al., 2020). Suicide is a major public health concern globally with nearly 800,000 deaths due to suicide worldwide (Ferrari et al., 2014; Vigo et al., 2016). It also affects people across the life course – children, adolescents, as well as older adults. Of all suicide deaths, 58% occur between the ages of 15–49 years which indicates the large magnitude of potentially productive years of life lost (Vos et al., 2020). In older age, major life events and complex transitions may be precipitants for suicide. According to GBD 2019 results, death rates from suicide are much higher in older age groups than in younger ones (24·53 per 100,000 for ages greater than 70 and 14·25 per 100,000 between 50 and 69 years vs 11·19 per 100,000 between 15 and 49 years). There are also differences in sex-specific suicide rates, with the suicide death rate among males being consistently higher than that of females over time (13·5 per 100,000 in males vs 6·1 in females in 2019) (Vos et al., 2020). Globally, India and China continue to report the highest number of deaths by suicide along with distinctly dominant suicide methods such as poisoning, hanging, and self-immolation (Ajdacic-Gross et al., 2008; Ferrari et al., 2014; James et al., 2018; Wu et al., 2012). This suggests a continued need to assess underlying risk factors and potential target areas for suicide prevention interventions in low and middle-income countries.

The risk of suicide attributable to mental disorders (MDs) is well established in the literature (Ferrari et al., 2014; Whiteford et al., 2013). Existing reviews indicate that suicide risk is particularly high at the time of diagnosis for affective disorders, and schizophrenia (Cavanagh et al., 2003; Harris and Barraclough, 1997; Inskip et al., 1998; Li et al., 2011; Walker et al., 2015). Up to 80% of suicide deaths have been attributed to a mental or substance use disorder in high income countries compared to approximately 70% in low income countries (Ferrari et al., 2014). However, the World Health Organization (WHO) classification followed by the GBD study captures suicide related deaths under ‘Injuries’ as the direct (rather than underlying) cause of death, while MDs are grouped separately within ‘Non-Communicable diseases (Ferrari et al., 2014). To estimate the proportion of suicide related deaths due to mental and substance use disorders (MSDs), previous work by Ferrari and collaborators applied comparative risk assessment (CRA) methodology to GBD 2010 data. They obtained RR estimates from a review of the literature which were then pooled and combined with GBD 2010 prevalence estimates to generate population-attributable fractions (PAFs). In their review of this literature, Ferrari and collaborators indicated that 62·2% of the burden estimated for suicide in an earlier iteration of the GBD study could be attributed to MSDs. MSDs moved from the 5th to the 3rd leading class of disease burden once suicide YLLs were reassigned to MSDs (Ferrari et al., 2014).

Given these findings, it is important to periodically re-assess the effect of MDs on premature mortality risk by updating the evidence base for the relationship between MDs and suicide. The work by Ferrari and Collaborators was conducted using GBD 2010 data and systematic literature reviews conducted in 2014. Analyses from this previous work largely comprised pooled RRs obtained using a simple meta-analytic approach. This approach did not account for sources of variation that may contribute to differences in reported suicide risk. These are important to consider in order to understand the impact of differences in study methods and subgroups on reported magnitude of suicide risk which, to date, remain largely unexplored. Existing reviews on this topic typically define broad inclusion criteria including vulnerable or high-risk subgroups (da Silva Costa et al., 2015; Rotenstein et al., 2016). These may introduce measurement error and subsequently result in potentially inflated risk estimates. The analytic approach in these reviews also typically involve pooled analyses that do not fully adjust for sources of measurement error (e.g. study design, sampling strategy, etc.) or assess the impact of other ecological variables (e.g. region or individual characteristics). This current systematic literature review aims to account for these limitations by providing updated suicide risk estimates adjusted in a meta-regression for key demographic and methodological covariates that may contribute to variation in study-reported estimates. We use the earlier review by Ferrari et al. (2014) as the basis and template for this work. We expand the search strings to incorporate self-harm and self-injury to maximize the scope of potentially eligible studies. Relevant data from 2010 to 2019 updates this systematic review, this the most comprehensive review of this risk-outcome pair to the best of our knowledge. This review will also contribute to measurement of mortality that can be indirectly attributed to mental disorders.

## Material and methods

### Case definition

The MDs included in this paper were those identified previously as risk factors for suicide (Vigo et al., 2016). They were defined according to the Diagnostic and Statistical Manual of Mental Disorders (DSM) or the International Classification of Diseases (ICD) diagnostic criteria (American Psychiatric Association, 1994; Fleischmann and de Leo, 2014; WHO, 2019). The mental disorders included were major depressive disorder (MDD), dysthymia, bipolar disorder, anxiety disorders, and schizophrenia. Suicide was defined as cases meeting criteria for ICD cause of death codes for intentional self-inflicted poisoning or injury.

### Systematic review and assessment of the literature

We expanded upon the 2014 systematic review by Ferrari and colleagues which covered the literature between 1966 and 2010 (See appendix). The search protocol adhered to the Preferred Reporting Items for Systematic Reviews and Meta-Analyses (PRISMA) guidelines and are described in the appendix (Moher et al., 2009). Three major scholarly databases, namely Medline, Embase, and PsychINFO were searched between January 2010 and June 2019 Studies were eligible for inclusion based on the following criteria:

(1) Considered MDs (as defined by DSM or ICD) as a risk factor associated with death by suicide
(2) Reported a relative risk (RR) with 95% uncertainty, or provided sufficient information for these to be calculated
(3) Were individual-level case-control or cohort studies defining a clear temporal association between the exposure (presence of mental disorder(s)) and outcome (suicide). Prospective cohort studies were considered the gold standard for inclusion. Studies using a cross-sectional design were excluded as this approach does not allow for a clear temporal association to be assessed.
(4) Study sample was representative of the general population of a given location (e.g.: community-based studies). Therefore, studies based on treated samples, linked psychiatric treatment registries, health insurance or claims data, or vulnerable populations (prisoners, pregnant women, veterans, homeless, etc.) were excluded.

In addition to the electronic database search, experts in the field were consulted and recently published reviews on this topic were reviewed for any additional data sources. All data from earlier reviews were first re-assessed for eligibility and accepted only if they met criteria for this review. Relevant information on effect sizes, study methodology and sample characteristics were extracted and compiled for each study. References were managed and duplicates were removed via EndNote citation management software. MM conducted the data searches and data extraction. AF and DS re-assessed study eligibility as needed and reconciled any questions around study inclusion.

### Statistical analysis

A multi-level meta-regression approach was used to obtain pooled relative risk estimates (RRs) and 95% uncertainty intervals for each disorder. We chose a multi-level meta-regression approach as it is best suited for data that are inherently nested or clustered within larger groups, as was the case here. Most studies in our dataset reported multiple effect sizes by disorder, age and/or sex and therefore were not independent observations. The multi-level approach with random effect terms for studies and disorder groups allowed us to account for any within and between study dependencies in the data at these levels. An unstructured variance-covariance matrix was used for random effects variances. The details and applications of this method are described in detail elsewhere (Assink and Wibbelink, 2016; Cheung, 2014). Exploratory forest plots for disorder-specific RRs and variance-covariance matrices of models with different correlation thresholds are reported in the appendix.

We made use of a series of covariates to quantify and adjust for (where appropriate) sources of heterogeneity in the data. A detailed explanation of covariates is provided in Table 1. The inclusion of covariates in the model occurred sequentially, starting with (Ajdacic-Gross et al., 2008) demographic variables known to be primary sources of variation (age, sex, disorder type, location) (Ali et al., 2016); methodological variables (follow-up time, sampling type, response rate, study design, estimate adjustment); and (American Psychiatric Association, 1994) the Sociodemographic Index (SDI) and the Health Access Quality Index (HAQI). Changes to effect-sizes due to the inclusion of new variables were reviewed at each step.

**Table 1:** List of covariates.

Covariate	Definition and Reference Levels	Details
Age	Mid-point of age range	MD prevalence and corresponding suicide risk are known to vary with age and by sex (Whiteford et al., 2015). Therefore, they are important sources of variation to consider in our analyses
Percent Female	Continuous covariate representing the proportion of females in study sample (Ranges from 0 to 1)	
Follow-up time	Follow-up time in years	Suicide is a relatively rare outcome compared to other causes of death. Therefore, the duration of follow-up may impact the number of study-reported outcomes
Response rate	Proportion of sample remaining after loss to follow-up/dropout	The response rate provides important information about possible selection bias in the sample.
Disorder	GBD mental disorder categories (Reference: MDD)	These are the primary risk factors for suicide being assessed. MDD was chosen as the reference since it was the most commonly assessed disorder among selected studies.
Estimate adjustment	Indicator for whether or not effect size has been adjusted for potential confounders such as individual demographics, socioeconomic status, family psychiatric history, etc. Reference: Adjusted for potential confounders	Study-reported effect sizes may be adjusted for potential confounders that are known to influence the MD-suicide association. These may be different from (and typically lower) than unadjusted effect sizes. Therefore, our analyses examine variation in suicide risk by testing this methodological covariate.
Psychological Autopsy (PA) Method	Indicator for whether or not data was collected using psychological autopsy – which involves collecting data from all available sources such as family informants, medical records, and healthcare providers. (Reference: PA not used)	This covariate was assessed because the psychological autopsy method involves data collection from informants and therefore is susceptible to biases in measurement of psychopathology, event recall, choice of appropriate control groups, etc. (Brent, 1989).
Study design	Prospective (Reference) or retrospective design	We expected variation in study quality and effect sizes based on the choice of study design. Therefore, this covariate was included to examine if study design influenced pooled RRs (Brent, 1989).
Sampling type	Random/other methods (multistage, cluster sampling)	We tested this covariate because we expected studies using random sampling to have less biased samples than studies using other methods (Lester and Stack, 1989).
SDI	Sociodemographic Index value (SDI): A summarized metric of a location's socio-demographic development on a scale of 0 (lowest) to 1 (highest).	Higher SDI and HAQI are associated with better health outcomes and lower premature mortality. Therefore, these were tested in the model to see if they had an impact on suicide risk.
HAQI	Health Access Quality Index value: A summarized metric of healthcare access and quality on a scale of 0 (worst) to 100 (best). More details on the construction of the HAQI can be found in elsewhere (Barber et al., 2017).	
High income locations	Locations that are classified as high-income as per World Bank income classification (Reference) vs other locations (Fantom and Serajuddin, 2016).	High-income locations are known to have better health outcomes and lower premature mortality than low and middle-income countries. Therefore, we tested this variable to see if it influenced resulting suicide risk estimates.

Due to considerable variation in the data collection and analytical methodology used between studies, several analytical choices were made in order to maximize data use. If studies reported odds ratios for suicide, these were converted to RRs using methods described in the appendix. Data from categories such as ‘mental disorders’, ‘mood and psychotic disorders’ or ‘mood and alcohol use disorders’ were not used as these very broad categories were less informative than more specific disorder categories. We considered the use of a location-level covariate using either GBD super-region classifications (Vos et al., 2020)or World Bank income level groups (Fantom and Serajuddin, 2016). However, only 6 out of 20 studies were from low or middle-income settings, which may have limited statistical power to detect a significant effect in our analysis. For this reason, a location covariate was not used in the final model. Covariates in our final model included age, sex, disorder type, estimate adjustment, use of psychological autopsy method, and study design.

In order to test if specific estimates had greater influence on pooled results compared to others, exploratory Baujat plots were generated (Baujat et al., 2002). These plots display the squared residuals on the X-axis and influence on the model on the Y-axis. Any estimates that had high residuals or high influence were determined to be outliers. Studies with outliers were re-assessed and those determined to have limitations in quality and/or measurement methods were excluded. Sensitivity analyses were conducted by assessing if overall results changed significantly upon removal of outliers as observed from the Baujat plots (Appendix). The distribution of variance across the levels included in our model was calculated using methods described in Cheung et al. (2014). This provided the proportion of the overall variance attributed to each level. All meta-regression analyses were conducted using the metafor package in R v3·5·2 (R Foundation for Statistical Computing, 2019; Viechtbauer, 2010).

## Results

**Fig. 1:**
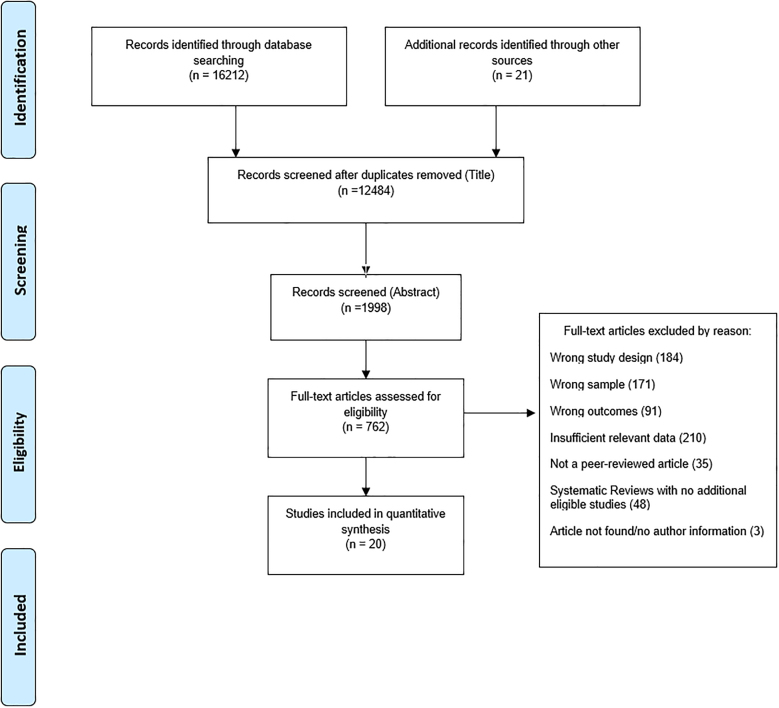
Study selection flowchart.

A total of 20 studies and 69 individual RRs were included in the final analysis (See Fig. 1). These studies covered 10 countries and 4 GBD regions. Anxiety disorders and MDD were the most commonly assessed MDs. (See Table 2 for a summary of selected studies). There was wide variation in reported sample sizes (84–68378) and follow-up time (1 year–64 years).

**Table 2:** List of selected studies.

Study	Region	Disorders included in Analysis
(Anderberg et al., 2016)	Western Europe	Anxiety Disorders
(Athey et al., 2018)	North America	Anxiety Disorders, Schizophrenia
(Brent et al., 1999)	North America	Anxiety Disorders
(Conwell et al., 2010)	North America	Major Depressive Disorders, Anxiety Disorders, Schizophrenia
(de Leo et al., 2013)	Australasia	Anxiety Disorders, Major Depressive disorders, Schizophrenia
(Giupponi et al., 2018)	Western Europe	Bipolar disorders, Major Depressive Disorders, Schizophrenia
(Hirokawa et al., 2012)	Asia-Pacific	Major Depressive Disorder, Anxiety Disorders, Schizophrenia, Dysthymia
(Holmstrand et al., 2015)	Western Europe	Major Depressive Disorders, Schizophrenia, Anxiety Disorders
(Lasserre et al., 2016)	Western Europe	Major Depressive Disorders
(Lesage et al., 1994)	North America	Major Depressive Disorder, Schizophrenia, Anxiety Disorders, Bipolar disorder
(Niu et al., 2018)	East Asia	Major Depressive Disorder
(Oğuzhanoğlu et al., 2018)	North Africa and Middle East	Major Depressive disorders, Schizophrenia
(Page et al., 2014)	Australasia	Anxiety Disorders
(Séguin et al., 2011)	North America	Anxiety disorders
(Shaffer et al., 1996)	North America	Major Depressive Disorders, Dysthymia, Schizophrenia, Bipolar Disorder, Anxiety Disorders,
(Shibre et al., 2014)	Sub-Saharan Africa	Schizophrenia, Bipolar Disorder, Major Depressive Disorders
(Tong and Phillips, 2010)	East Asia	Anxiety Disorders, Schizophrenia
(Waern, 2003)	Western Europe	Major Depressive Disorders, Anxiety disorders
(Zhang et al., 2010)	East Asia	Anxiety Disorders, Bipolar Disorders, Dysthymia, Major Depressive disorders, Schizophrenia
(Zhou et al., 2019)	East Asia	Anxiety Disorders, Bipolar Disorders, Dysthymia, Major Depressive disorders, Schizophrenia

### Multi-level meta-regression

Table 3 presents the meta-regression model coefficients and Table 4 presents the predicted RRs adjusted for select covariates. Disorder type, sex, age, study design, estimate adjustment, and use of psychological autopsy (PA) were tested in the model for suicide risk (Table 3). The amount of the overall variance attributed to random effects was 73.14%. We found no significant associations between suicide risk and response rate, follow-up time, sampling type, high vs low/middle-income locations, SDI, or HAQI. These variables were therefore not included in the final model. Our sensitivity analyses also showed no major change in significance and direction of estimates upon removal of outliers (4 observations – see appendix).

**Table 3:** Meta-regression model coefficients by covariate.

Covariates		Coefficient	P Value
Cochran's test for residual heterogeneity		QE (Walker et al., 2015) = 177·98	<0·0001
QM-Test of moderators		QM (Baujat et al., 2002) = 68·96	<0·0001
Age	NA	0·0053	0·0094
Both	11 studies/39 observations	Reference category	
Percent Female	Male: 7 studies/18 observations	−0·0346	0·7518
	Female: 6 studies/12 observations		
**Disorder**			
Major Depressive Disorders	14 studies/17 observations	Reference category	
Anxiety Disorders	17 studies/23 observations	−0·446	<0·0001
Bipolar disorder	7 studies/7 observations	−0·234	0·0217
Dysthymia	4 studies/4 observations	−0·62	0·0011
Schizophrenia	13 studies/18 observations	−0·245	0·0006
**Estimate adjustment**			
Adjusted for confounders	5 studies/15 observations	Reference category	
Unadjusted for confounders	15 studies/54 observations	−0·35	0·0672
**Study Design**			
Prospective design	4 studies/10 observations	Reference category	
Retrospective design	16 studies/59 observations	−0·932	0·0035
**Use of Psychological Autopsy**			
Psychological autopsy not used	2 studies/4 observations	Reference category	
Psychological autopsy used	18 studies/65 observations	−0·0008	0·99

**Table 4:** Predicted relative risks for suicide.

Disorder	Both	Males	Females
Major Depressive Disorder	7·64 [4·3, 13·58]	7·78 [4·34, 13·93]	7·51 [4·18, 13·51]
Dysthymia	4·11 [2·09, 8·09]	4·18 [2·12, 8·26]	4·04 [2·02, 8·06]
Anxiety Disorders	4·89 [2·76, 8·69]	4·98 [2·78, 8·91]	4·81 [2·68, 8·64]
Bipolar Disorder	6·05 [3·38, 10·83]	6·15 [3·4, 11·13]	5·94 [3·29, 10·75]
Schizophrenia	5·98 [3·33, 10·72]	6·09 [3·73, 10·98]	5·88 [3·24, 10·66]

Age was associated with an increase in suicide risk (Table 3). Among methodological covariates, the use of retrospective study design was associated with lower suicide risk compared to prospective study design. There was no significant effect of estimate adjustment or use of the psychological autopsy method.

Suicide risk was significantly associated with the presence of MDD, anxiety disorders, bipolar disorder, dysthymia, and schizophrenia (Table 3). Predicted RRs were the highest for MDD, bipolar disorder, and schizophrenia (Table 4). RRs for females were consistently lower than RRs for males across all disorders (although this association was not significant).

RRs adjusted for sex, age, disorder, study design, estimate adjustment, and psych-autopsy method.

## Discussion

Our results confirm that MDs are important risk factors for suicide. This association remained after adjusting for methodological factors. The earlier Ferrari et al. (2014) review provided pooled estimates of suicide risk for each disorder obtained via a random effects meta-analytic approach. This updated review improves upon this approach by using a multi-level meta-regression model with predictive covariates that can (Ajdacic-Gross et al., 2008) better utilize (in its nested approach) all the data available across studies and (Ali et al., 2016) more robustly explain the heterogeneity in the data reported between studies to separate measurement error from variability that is ‘real’ and due to ecological variables.

Our findings showed that MDD, dysthymia, anxiety disorders, bipolar disorder, and schizophrenia were significantly associated with suicide risk. The selection of disorders attaining statistical significance, are consistent with meta-analytic results from other studies exploring suicide mortality due to MDs (Baxter et al., n.d.; Chesney et al., 2014; Darvishi et al., 2015; Hawton et al., 2013). An earlier review on anxiety as a risk factor for suicide did not find anxiety disorders to be an important predictor of death by suicide (Bentley et al., 2016). This may have been due to the inclusion of studies that used symptom scales to assess anxiety disorders – which are best used for screening or symptomatic evaluation rather than provide a clinical diagnosis. Secondly, the inclusion of any individual anxiety diagnosis may have attenuated the pooled risk of suicide. Reviews may also focus only on specific disorders (da Silva Costa et al., 2015). Although these provide an important profile of disorder-specific suicide risk, they exclude psychiatric comorbidity - an important contributor to and perhaps catalyst for suicide risk. Reviews may also draw from national treatment registers for data from samples with mental disorders (San Too et al., 2019). National treatment registers are a useful source of data on mental disorders often drawn from large samples and allow for a more efficient study of rare outcomes such as death by suicide. However, it is important to consider that this mode of data collection may also be inherently biased as it does not capture mental disorder cases among those who may not have made contacts with any health service. It is likely that register-based diagnoses may reflect a higher severity of mental disorders than what may be present in the general population. Therefore, the use of registry-based data is expected to be associated with an increase in or overestimation of pooled estimates of suicide risk. Some reviews also examine suicidal behavior (such as suicide attempts and/or self-injury with an intent to die). A recent review by Gili and colleagues examine the evidence for mental and substance use disorders as risk factors for suicidal behavior (suicidal acts and attempts) among young people (Gili et al., 2019). Although this review provides important insights into the risk for suicide attempts that frequently precede suicide deaths in younger populations, the analyses use ORs instead of RRs and use broad groups of disorders which may limit interpretation for disorder-specific risks especially for depressive disorders which are known to account for a large proportion of suicide risk. It is important to highlight that the disorder-specific pooled RRs obtained here are considerably lower than those found in the earlier review by Ferrari et al. (2014). The use of demographic, methodological, and location-level covariates may have contributed to this difference. We adjusted for study-level random effects using a hierarchical approach that is also likely to yield lower adjusted RRs for suicide compared to a standard meta-analytic approach that has typically been used to generate pooled effect sizes in the past. We also used stricter inclusion criteria and data prepping procedures than what has been used in the existing literature. Furthermore, meta-analytical studies will often use odds ratios as an approximation for RRs if the prevalence of suicide is known to be very low. This may well overestimate RRs and lead to higher effect sizes we usually see in other studies (Walker et al., 2015). This study attempts to take suicide risk measurement a step further by including covariates that account for measurement error. Although uncertainty in the model remains, we believe that the estimates are more robust as a result of this.

In our model, older age was associated with higher RRs. This is consistent with findings in the literature where older age is likely to be associated with an increased risk of suicide ((Whiteford et al., 2015). Among methodological variables, the use of a retrospective study design was associated with lowered suicide risk. It is possible that a prospective study design is better at establishing a temporal association between mental disorders and suicide which may not be possible in a retrospective design. Additionally, studies using a retrospective design may not be able to capture the impact of confounders on mental disorders and suicide as accurately as those using a prospective design.

We found no significant associations between the use of psychological autopsy and suicide risk. This may be due to the low number of non-psych autopsy studies that were included in the analyses. Overestimation or underestimation of suicide risk in psych-autopsy studies may be due to biases in how psychopathology is measured, specifically the choice of informants, choice of comparison group, and lowered event recall - especially if the assessment is conducted much later than the date of suicide. Although our study did not empirically assess these components beyond a dichotomous measure of psychological autopsy use, it is reasonable to assume that they may contribute to the heterogeneity in reported suicide risk beyond what we found in our analyses.

Adjusted RRs were not significantly different from unadjusted RRs in our study. However, it was important to include this covariate in our model since RRs that controlled for known potential confounders were likely be more accurate in estimating true suicide risk compared to crude RRs. A possible reason for not detecting a significant effect may be the low number of studies adjusted RRs. There is considerable variation in the number and type of confounders controlled for between studies. Some studies may control for a single confounder (such as age, or educational attainment). Other studies may control for a larger number and range of confounders such as psychiatric comorbidity, family history of MDs, socioeconomic status, physical illnesses, and others. Although our current analyses did not empirically assess the impact of this variation on suicide risk, it is plausible that studies that control for as many potential confounders as possible likely report estimates of a higher quality and reliability compared to studies that do not.

We were unable to empirically assess location-level differences in RRs due to the lack of sufficient studies from LMICs. Out of 20 studies, 14 were from high-income locations - mostly Western Europe and North America. Given the lack of income level-specific estimates across disorders, the uncertainty across the results produced is greater and interpretability of the resulting sex- and region-specific RR are lowered. The sparsity of data from LMICs may be due to variation and constraints in country-level reporting of suicide deaths. Suicide continues to be a criminal offence in many countries in Africa, Asia, and South America, which potentially leads to systematic underreporting of suicide cases (de Leo et al., 2013; Fleischmann and de Leo, 2014; Khan, 2005). The global data coverage for MDs is also low for LMICs(Baxter et al., 2013a, Baxter et al., 2013b). Better quality data from LMICs along with more sex-specific data on MDs and suicide may allow us to make more substantive conclusions about regional and sex-specific patterns in RRs for suicide.

This systematic review provides a useful insight into the recent available evidence on the risk of suicide among those with versus without MDs and the heterogeneity that may exist in the data based on study-specific attributes. Our findings of MDs being significant predictors of suicide risk are largely consistent with findings from other individual-level and meta-analytic studies (da Silva Costa et al., 2015; Rotenstein et al., 2016; Walker et al., 2015). Based on the GBD study, MDs are the some of the leading causes of non-fatal disability (Whiteford et al., 2015, 2013). However, very few MDs are attributed as direct causes of suicide despite overwhelming evidence in the literature. Findings from this study may better inform the burden from premature mortality directly attributed to MDs in GBD.

These findings also support the need for adequate mental healthcare as an important suicide prevention measure. Research using World Mental Health surveys shows that a large treatment gap exists for those exhibiting suicidal ideation and behaviors. Only 39% of those with suicidality receive any form of treatment worldwide. This proportion is even lower in low-income countries where only 17% of those with suicidality receive any form of treatment (Bruffaerts et al., 2011). Although there exists evidence in support of select interventions for suicide prevention, improving prevention efforts at earlier stages of care may increase the likelihood of fulfilling this unmet need for care (Mann et al., 2005). These findings also emphasize the continued need for primary interventions for suicide prevention that focus on adequate screening for and detection of MDs in different contexts such as community, primary care, and school-based settings (Ali et al., 2016; Bruffaerts et al., 2011; Dowdy et al., 2015; Guntuku et al., 2017; Kline and Schiffman, 2014; Siu et al., 2016). However, precision in MD detection is dependent on the screening tool and classification thresholds used - particularly in varying cultural contexts. Arango and colleagues define secondary and tertiary prevention efforts in addition to primary prevention efforts in their review (Arango et al., 2018). Secondary prevention efforts that focus on reducing the incidence of MDs and tertiary prevention efforts that treat those with established MDs are important as they address the role of MDs in suicide prevention (Arango et al., 2018). A systematic review on suicide prevention strategies found that in addition to direct suicide prevention strategies such as restricting access to lethal means, pharmacological and psychological treatment of MDs are also important in suicide prevention (Zalsman et al., 2016).

Several limitations constrained our analyses. First, the geographic representativeness of our analysis is limited. Although our inclusion and exclusion criteria ensured the selection of high quality studies, these were mostly from high-income countries in North America and Western Europe. Therefore, studies from low and middle-income countries are under-represented in this analysis indicating the need for more published data from these regions. Second, we were unable to provide estimates for additional psychiatric disorders such as eating disorders due to the lack of studies that fulfilled our selection criteria. Third, we acknowledge that the uncertainty intervals for predicted RRs were at times wide and overlapping between disorders due to lack of data for some disorders and by income-level. Fourth, analyzing data for all available MDs in a single meta-regression model may either attenuate or amplify the magnitude of disorder-specific RRs. Fifth, the standard meta-regression framework allows for uncertainty intervals of predicted RRs to capture error attributed to fixed effects only and not between-study heterogeneity. Sixth, the scope of this analysis was confined to measuring the risk of suicide deaths only. While suicidal ideation and attempts are important precursors to death by suicide, our study did not explore these outcomes as they may have different risk profiles compared to completed suicides. Lastly, although we tried to quantify as many methodological sources of variation as possible, we were unable to account for all sources of measurement error. We excluded methodological covariates such as sampling type, follow-up duration, and response rate as there was insufficient power to detect an effect.

## Conclusion

MDs are associated with an increased risk for suicide. This synthesized analysis shows that the magnitude of study-reported risk may vary depending on study methodology and disorders examined. Therefore, study quality and choice of methods are important to consider in public reports on aggregated evidence. More research is needed to better quantify evidence for MD burden and suicide risk with higher precision in low and middle-income settings. These efforts will assist policy makers in framing evidence-based suicide prevention strategies and make mental health care an important part of their agendas.

## Funding

This work was supported by the 10.13039/100000865Bill and Melinda Gates Foundation, Queensland 10.13039/501100003921Department of Health, and the 10.13039/501100000925National Health and Medical Research Council Early Career Fellowship Grant APP1121516.

## Role of the funding source

The funders had no role in study design, data collection, data analysis, data interpretation, or writing of the report. The corresponding author had full access to all the data in the study and had final responsibility for the decision to submit for publication.

## CRediT authorship contribution statement

**Modhurima Moitra:** Conceptualization, Methodology, Software, Formal analysis, Data curation, Writing – original draft, Visualization. **Damian Santomauro:** Conceptualization, Methodology, Software, Validation, Formal analysis, Writing – review & editing, Supervision. **Louisa Degenhardt:** Resources, Data curation, Writing – review & editing. **Pamela Y. Collins:** Writing – review & editing, Supervision. **Harvey Whiteford:** Writing – review & editing. **Theo Vos:** Writing – review & editing. **Alize Ferrari:** Conceptualization, Methodology, Validation, Writing – review & editing, Supervision.

## Declaration of competing interest

We declare no competing interests.

## References

[bib1] Ajdacic-Gross V., Weiss M.G., Ring M., Hepp U., Bopp M., Gutzwiller F., Rössler W. (2008). Methods of suicide: international suicide patterns derived from the WHO mortality database. Bull. World Health Organ..

[bib2] Ali G.-C., Ryan G., de Silva M.J. (2016). Validated screening tools for common mental disorders in low and middle income countries: a systematic review. PloS One.

[bib3] American Psychiatric Association A.P. (1994). Diagnostic and Statistical Manual of Mental Disorders (DSM-IV).

[bib4] Anderberg J., Bogren M., Mattisson C., Brådvik L. (2016). Long-term suicide risk in anxiety—the Lundby study 1947–2011. Arch. Suicide Res..

[bib5] Arango C., Díaz-Caneja C.M., McGorry P.D., Rapoport J., Sommer I.E., Vorstman J.A., McDaid D., Marín O., Serrano-Drozdowskyj E., Freedman R. (2018). Preventive strategies for mental health. Lancet Psychiatr..

[bib6] Assink M., Wibbelink C.J.M. (2016). Fitting three-level meta-analytic models in R: a step-by-step tutorial. Quant. Methods Psychol..

[bib7] Athey A., Overholser J., Bagge C., Dieter L., Vallender E., Stockmeier C.A. (2018). Risk-taking behaviors and stressors differentially predict suicidal preparation, non-fatal suicide attempts, and suicide deaths. Psychiatr. Res..

[bib8] Barber R.M., Fullman N., Sorensen R.J.D., Bollyky T., McKee M., Nolte E., Abajobir A.A., Abate K.H., Abbafati C., Abbas K.M. (2017). Healthcare Access and Quality Index based on mortality from causes amenable to personal health care in 195 countries and territories, 1990–2015: a novel analysis from the Global Burden of Disease Study 2015. Lancet.

[bib9] Baujat B., Mahé C., Pignon J., Hill C. (2002). A graphical method for exploring heterogeneity in meta‐analyses: application to a meta‐analysis of 65 trials. Stat. Med..

[bib10] Baxter A.J., Patton G., Scott K.M., Degenhardt L., Whiteford H.A. (2013). Global epidemiology of mental disorders: what are we missing?. PloS One.

[bib11] Baxter A.J., Scott K.M., Vos T., i Whiteford n.d., Ha (2013). Global prevalence of anxiety disorders: a systematic review and meta-regression. Psychol. Med..

[bib12] Bentley K.H., Franklin J.C., Ribeiro J.D., Kleiman E.M., Fox K.R., Nock M.K. (2016). Anxiety and its disorders as risk factors for suicidal thoughts and behaviors: a meta-analytic review. Clin. Psychol. Rev..

[bib13] Brent D.A. (1989). The psychological autopsy: methodological considerations for the study of adolescent suicide. Suicide Life-Threatening Behav..

[bib14] Brent D.A., Baugher M., Bridge J., Chen T., Chiappetta L. (1999). Age-and sex-related risk factors for adolescent suicide. J. Am. Acad. Child Adolesc. Psychiatry.

[bib15] Bruffaerts R., Demyttenaere K., Hwang I., Chiu W.-T., Sampson N., Kessler R.C., Alonso J., Borges G., de Girolamo G., de Graaf R. (2011). Treatment of suicidal people around the world. Br. J. Psychiatr..

[bib16] Cavanagh J.T.O., Carson A.J., Sharpe M., Lawrie S.M. (2003). Psychological autopsy studies of suicide: a systematic review. Psychol. Med..

[bib17] Chesney E., Goodwin G.M., Fazel S. (2014). Risks of all‐cause and suicide mortality in mental disorders: a meta‐review. World Psychiatr..

[bib18] Cheung M.W.-L. (2014). Modeling dependent effect sizes with three-level meta-analyses: a structural equation modeling approach. Psychol. Methods.

[bib19] Conwell Y., Duberstein P.R., Hirsch J.K., Conner K.R., Eberly S., Caine E.D. (2010). Health status and suicide in the second half of life. Int. J. Geriatr. Psychiatr.: J. Psychiatr. Late Life Allied Sci..

[bib20] da Silva Costa L., Alencar Á.P., Neto P.J.N., dos Santos M., do S.V., da Silva C.G.L., Pinheiro S. de F.L., Silveira R.T., Bianco B.A.V., Júnior R.F.F.P., de Lima M.A.P. (2015). Risk factors for suicide in bipolar disorder: a systematic review. J. Affect. Disord..

[bib21] Darvishi N., Farhadi M., Haghtalab T., Poorolajal J. (2015). Alcohol-related risk of suicidal ideation, suicide attempt, and completed suicide: a meta-analysis. PloS One.

[bib22] de Leo D., Draper B.M., Snowdon J., Kõlves K. (2013). Suicides in older adults: a case–control psychological autopsy study in Australia. J. Psychiatr. Res..

[bib23] Dowdy E., Furlong M., Raines T.C., Bovery B., Kauffman B., Kamphaus R.W., Dever B. v, Price M., Murdock J. (2015). Enhancing school-based mental health services with a preventive and promotive approach to universal screening for complete mental health. J. Educ. Psychol. Consult..

[bib24] Fantom N., Serajuddin U. (2016). The World Bank's Classification of Countries by Income.

[bib25] Ferrari A.J., Norman R.E., Freedman G., Baxter A.J., Pirkis J.E., Harris M.G., Page A., Carnahan E., Degenhardt L., Vos T. (2014). The burden attributable to mental and substance use disorders as risk factors for suicide: findings from the Global Burden of Disease Study 2010. PloS One.

[bib26] Fleischmann A., de Leo D. (2014). The World Health Organization's Report on Suicide: a Fundamental Step in Worldwide Suicide Prevention.

[bib27] Gili M., Castellví P., Vives M., de la Torre-Luque A., Almenara J., Blasco M.J., Cebrià A.I., Gabilondo A., Pérez-Ara M.A., Miranda-Mendizabal A. (2019). Mental disorders as risk factors for suicidal behavior in young people: a meta-analysis and systematic review of longitudinal studies. J. Affect. Disord..

[bib28] Giupponi G., Innamorati M., Baldessarini R.J., de Leo D., de Giovannelli F., Pycha R., Conca A., Girardi P., Pompili M. (2018). Factors associated with suicide: case-control study in South tyrol. Compr. Psychiatr..

[bib29] Guntuku S.C., Yaden D.B., Kern M.L., Ungar L.H., Eichstaedt J.C. (2017). Detecting depression and mental illness on social media: an integrative review. Curr. Opinion Behav. Sci..

[bib30] Harris E.C., Barraclough B. (1997). Suicide as an outcome for mental disorders. A meta-analysis. Br. J. Psychiatr..

[bib31] Hawton K., i Comabella C.C., Haw C., Saunders K. (2013). Risk factors for suicide in individuals with depression: a systematic review. J. Affect. Disord..

[bib32] Hirokawa S., Kawakami N., Matsumoto T., Inagaki A., Eguchi N., Tsuchiya M., Katsumata Y., Akazawa M., Kameyama A., Tachimori H. (2012). Mental disorders and suicide in Japan: a nation-wide psychological autopsy case–control study. J. Affect. Disord..

[bib33] Holmstrand C., Bogren M., Mattisson C., Brådvik L. (2015). Long‐term suicide risk in no, one or more mental disorders: the Lundby Study 1947–1997. Acta Psychiatr. Scand..

[bib34] Inskip H., Harris C., Barraclough B. (1998). Lifetime risk of suicide for affective disorder, alcoholism and schizophrenia. Br. J. Psychiatr..

[bib35] James S.L., Abate D., Abate K.H., Abay S.M., Abbafati C., Abbasi N., Abbastabar H., Abd-Allah F., Abdela J., Abdelalim A. (2018). Global, regional, and national incidence, prevalence, and years lived with disability for 354 diseases and injuries for 195 countries and territories, 1990–2017: a systematic analysis for the Global Burden of Disease Study 2017. Lancet.

[bib36] Khan M.M. (2005). Suicide prevention and developing countries. J. R. Soc. Med..

[bib37] Kline E., Schiffman J. (2014). Psychosis risk screening: a systematic review. Schizophr. Res..

[bib38] Lasserre A.M., Marti-Soler H., Strippoli M.-P.F., Vaucher J., Glaus J., Vandeleur C.L., Castelao E., Marques-Vidal P., Waeber G., Vollenweider P. (2016). Clinical and course characteristics of depression and all-cause mortality: a prospective population-based study. J. Affect. Disord..

[bib39] Lesage A.D., Boyer R., Grunberg F., Vanier C., Morissette R., Ménard-Buteau C., Loyer M. (1994). Suicide and mental disorders: a case-control study of young men. Am. J. Psychiatr..

[bib40] Lester D., Stack S. (1989). Bias resulting from the choice of sample and results of cross-national analyses of suicide rates. Qual. Quantity.

[bib41] Li Z., Page A., Martin G., Taylor R. (2011). Attributable risk of psychiatric and socio-economic factors for suicide from individual-level, population-based studies: a systematic review. Soc. Sci. Med..

[bib42] Mann J.J., Apter A., Bertolote J., Beautrais A., Currier D., Haas A., Hegerl U., Lonnqvist J., Malone K., Marusic A. (2005). Suicide prevention strategies: a systematic review. Jama.

[bib43] Moher D., Liberati A., Tetzlaff J., Altman D.G. (2009). Academia and clinic annals of internal medicine preferred reporting items for systematic reviews and meta-analyses: the PRISMA statement. Ann. Intern. Med..

[bib44] Niu L., Jia C., Ma Z., Wang G., Yu Z., Zhou L. (2018). Validating the Geriatric Depression Scale with proxy-based data: a case-control psychological autopsy study in rural China. J. Affect. Disord..

[bib45] Oğuzhanoğlu N.K., Uğurlu T.T., Acar K., Atesci F. (2018). A psychological and social perspective on completed suicides in western Anatolia, Turkey: a case-control psychological autopsy study. Düşünen Adam J. Psychiatry Neurol. Sci..

[bib46] Page A., Morrell S., Hobbs C., Carter G., Dudley M., Duflou J., Taylor R. (2014). Suicide in young adults: psychiatric and socio-economic factors from a case–control study. BMC Psychiatr..

[bib47] R Foundation for Statistical Computing (2019). R: A Language and Environment for Statistical Computing.

[bib48] Rotenstein L.S., Ramos M.A., Torre M., Segal J.B., Peluso M.J., Guille C., Sen S., Mata D.A. (2016). Prevalence of depression, depressive symptoms, and suicidal ideation among medical students: a systematic review and meta-analysis. Jama.

[bib49] San Too L., Spittal M.J., Bugeja L., Reifels L., Butterworth P., Pirkis J. (2019). The association between mental disorders and suicide: a systematic review and meta-analysis of record linkage studies. J. Affect. Disord..

[bib50] Séguin M., Renaud J., Lesage A., Robert M., Turecki G. (2011). Youth and young adult suicide: a study of life trajectory. J. Psychiatr. Res..

[bib51] Shaffer D., Gould M.S., Fisher P., Trautman P., Moreau D., Kleinman M., Flory M. (1996). Psychiatric diagnosis in child and adolescent suicide. Arch. Gen. Psychiatr..

[bib52] Shibre T., Hanlon C., Medhin G., Alem A., Kebede D., Teferra S., Kullgren G., Jacobsson L., Fekadu A. (2014). Suicide and suicide attempts in people with severe mental disorders in Butajira, Ethiopia: 10 year follow-up of a population-based cohort. BMC Psychiatr..

[bib53] Siu A.L., Bibbins-Domingo K., Grossman D.C., Baumann L.C., Davidson K.W., Ebell M., García F.A.R., Gillman M., Herzstein J., Kemper A.R. (2016). Screening for depression in adults: US preventive services task force recommendation statement. Jama.

[bib54] Tong Y., Phillips M.R. (2010). Cohort-specific risk of suicide for different mental disorders in China. Br. J. Psychiatr..

[bib55] Viechtbauer W. (2010). Conducting meta-analyses in R with the metafor package. J. Stat. Software.

[bib56] Vigo D., Thornicroft G., Atun R. (2016). Estimating the true global burden of mental illness. Lancet Psychiatr..

[bib57] Vos T., Lim S.S., Abbafati C., Abbas K.M., Abbasi M., Abbasifard M., Abbasi-Kangevari M., Abbastabar H., Abd-Allah F., Abdelalim A. (2020). Global burden of 369 diseases and injuries in 204 countries and territories, 1990–2019: a systematic analysis for the Global Burden of Disease Study 2019. Lancet.

[bib58] Waern M. (2003). Alcohol dependence and misuse in elderly suicides. Alcohol Alcohol.

[bib59] Walker E.R., McGee R.E., Druss B.G. (2015). Mortality in mental disorders and global disease burden implications: a systematic review and meta-analysis. JAMA Psychiatr..

[bib60] Whiteford H.A., Degenhardt L., Rehm J., Baxter A.J., Ferrari A.J., Erskine H.E., Charlson F.J., Norman R.E., Flaxman A.D., Johns N. (2013). Global burden of disease attributable to mental and substance use disorders: findings from the Global Burden of Disease Study 2010. Lancet.

[bib61] Whiteford H.A., Ferrari A.J., Degenhardt L., Feigin V., Vos T. (2015). The global burden of mental, neurological and substance use disorders: an analysis from the Global Burden of Disease Study 2010. PloS One.

[bib62] WHO (2019). ICD-10 Version:2019.

[bib63] Wu K.C.-C., Chen Y.-Y., Yip P.S.F. (2012). Suicide methods in Asia: implications in suicide prevention. Int. J. Environ. Res. Publ. Health.

[bib64] Zalsman G., Hawton K., Wasserman D., van Heeringen K., Arensman E., Sarchiapone M., Carli V., Höschl C., Barzilay R., Balazs J. (2016). Suicide prevention strategies revisited: 10-year systematic review. Lancet Psychiatr..

[bib65] Zhang J., Wieczorek W., Conwell Y., Tu X.-M., Wu B.-W., Xiao S., Jia C. (2010). Characteristics of young rural Chinese suicides: a psychological autopsy study. Psychol. Med..

[bib66] Zhou L., Wang G., Jia C., Ma Z. (2019). Being left-behind, mental disorder, and elderly suicide in rural China: a case–control psychological autopsy study. Psychol. Med..

