# Supplementary material for: Estimating the risk of suicide associated with mental disorders: A systematic review and meta-regression analysis
Source: J Psychiatr Res. 2021 May;137:242–9. doi: 10.1016/j.jpsychires.2021.02.053 (PMC8095367; doi:10.1016/j.jpsychires.2021.02.053)
Supplement: Multimedia component 1 [file mmc1.docx]

APPENDIX

**Search String:**

((((((((((((((("suicid*"[Title/Abstract] OR "self-harm"[Title/Abstract] OR "suicid*"[MeSH Terms] OR "self-harm"[MeSH Terms]))))

AND

("cohort"

OR "case control"

OR "case-control"

OR "autopsy")

AND

(("mood disorder*"[Title/Abstract]

OR "depress*"[Title/Abstract]

OR "dysthymi*"[Title/Abstract]

OR "bipolar"[Title/Abstract]

OR "manic"[Title/Abstract]

OR "mania"[Title/Abstract]

OR "Mood disorders"[MeSH Terms]

OR "Depressive disorders"[MeSH Terms]

OR "Depressive disorder, Major"[MeSH Terms]

OR "Bipolar disorder"[MeSH Terms]

OR "Dysthymic Disorders"[MeSH Terms]

OR "generalized anxiety disorder*"[Title/Abstract]

OR "post traumatic stress disorder*"[Title/Abstract]

OR "posttraumatic stress disorder*"[Title/Abstract]

OR "traumatic stress disorder*"[Title/Abstract]

OR "anxiety disorder*"[Title/Abstract]

OR "Anxiety Disorders"[Mesh: No Expansion]

OR "Schizo*"[Title/Abstract]

OR "Psychosis"[Title/Abstract]

OR "Psychotic"[Title/Abstract]

OR "Psychotic disorder"[MeSH Terms]

OR "Psychotic disorders"[MeSH Terms]

OR "Schizophrenia"[Mesh: No Expansion]

OR "Personality disorder"[Mesh: No Expansion]

OR "Anorexia*"[Title/Abstract]

OR "Bulimia*"[Title/Abstract]

OR "Eating disorder*"[Title/Abstract]

OR "binge eating disorder"[Title/Abstract]

OR "Binge eating disorder"[MeSH Terms]

OR "Anorexia nervosa"[MeSH Terms]

OR "Bulimia nervosa"[MeSH Terms]

OR "Eating disorder"[MeSH Terms]

OR ("alcohol*"[Title/Abstract] AND ("abus*"[Title/Abstract] OR "dependen*"[Title/Abstract] OR "misus*"[Title/Abstract] OR "addict*"[Title/Abstract]))

OR ("drug"[Title/Abstract] OR "substance"[Title/Abstract]) AND ("abus*"[Title/Abstract] OR "dependen*"[Title/Abstract] OR "addict*"[Title/Abstract] OR "misus*"[Title/Abstract])

OR "Alcohol-Related Disorder"[Mesh: No Expansion ]

OR "Alcohol-Induced Disorder"[Mesh: No Expansion]

OR "Fetal alcohol spectrum disorder"[MeSH Terms]

OR "Fetal alcohol disorder"[MeSH Terms]

OR "Alcoholism"[MeSH Terms]

OR "Amphetamine-related disorders"[MeSH Terms]

OR "Cocaine-related disorders"[MeSH Terms]

OR "Opioid-related disorders"[MeSH Terms]

OR "Substance-related disorders"[Mesh: No Expansion]

OR "opioid*"[Title/Abstract]

OR "heroin"[Title/Abstract]

OR "Cocaine"[Title/Abstract]

OR "Marijuana"[Title/Abstract]

OR "Cannabis"[Title/Abstract]

OR "Amphetamin*"[Title/Abstract]

OR "Methamphetam*"[Title/Abstract])))))))))))

**Inclusion/Exclusion Criteria:**

1. **Study Representativeness**: Studies using samples that are demographically representative of the community, city, region, or country of interest will be included. Therefore, estimates of suicide risk obtained from samples of minority groups or clinical trials will not be included.
2. **Timeframe**: Studies published between 2010 and 2019 (June) are included.
3. **Case Definitions**:
   - 1. Suicide: Defined as cases meeting the ICD-10 cause of death codes for intentional self-inflicted poisoning or injury (X60–X84)
     2. Mental Disorders: Studies that adhere to the definition of mental disorders as determined by the International classification of diseases (ICD-10) or the Diagnostic and Statistical Manual of Mental Disorders (DSM-III, IV, V) will be included. Risk estimates for suicide derived from subthreshold cases will not be included.
4. **Risk of Suicide** attributable to Mental Disorders: Studies must report effect sizes clearly quantifying the risk of suicide that can be attributable to one or more mental disorders or provide enough data to calculate effect sizes. This review will exclude studies that only report the risk of suicide attributable to causes unrelated to mental disorders.

**Data Extraction:**

- Information pertaining to study methodology, sample, cases, and effect size values will be extracted from data sources that satisfy our inclusion criteria.
- Aggregated estimates of risk of suicide attributed to mental disorders will be extracted if reported or calculated if sufficient data are available for calculations.
- If studies report suicide risk estimates by disorder type, severity, age-group, sex-group, or any other category, this information was extracted for further disaggregation of suicide risk where possible.
- If studies report odds ratios, these will be converted to relative risks using methods described in Barendregt 2010 (ref).
- If studies do not report mental disorder prevalence in the entire sample, year-location-sex specific prevalence estimates from the Global Burden of Disease will be used to calculate relative risks.
- An estimate of uncertainty (either a standard error or 95% uncertainty interval) around the risk estimate will be extracted if reported, or calculated using the formula:

$SE= \sqrt{2.1\left( \frac{P\left( 1-P \right)}{N} \right)}$

Where N=sample size, P= proportion/quantity of interest

**PRISMA checklist for the literature search to identify relative-risk estimates**

| **Section/topic** | **#** | **Checklist item** | **Reported on page #** |
| --- | --- | --- | --- |
| **TITLE** | | |  |
| Title | 1 | Identify the report as a systematic review, meta-analysis, or both. | 1 |
| **ABSTRACT** | | |  |
| Structured summary | 2 | Provide a structured summary including, as applicable: background; objectives; data sources; study eligibility criteria, participants, and interventions; study appraisal and synthesis methods; results; limitations; conclusions and implications of key findings; systematic review registration number. | 1 |
| **INTRODUCTION** | | |  |
| Rationale | 3 | Describe the rationale for the review in the context of what is already known. | 2 |
| Objectives | 4 | Provide an explicit statement of questions being addressed with reference to participants, interventions, comparisons, outcomes, and study design (PICOS). | 2-3 |
| **METHODS** | | |  |
| Protocol and registration | 5 | Indicate if a review protocol exists, if and where it can be accessed (e.g., Web address), and, if available, provide registration information including registration number. | N/A |
| Eligibility criteria | 6 | Specify study characteristics (e.g., PICOS, length of follow-up) and report characteristics (e.g., years considered, language, publication status) used as criteria for eligibility, giving rationale. | 3 |
| Information sources | 7 | Describe all information sources (e.g., databases with dates of coverage, contact with study authors to identify additional studies) in the search and date last searched. | 3 |
| Search | 8 | Present full electronic search strategy for at least one database, including any limits used, such that it could be repeated. | Appendix |
| Study selection | 9 | State the process for selecting studies (i.e., screening, eligibility, included in systematic review, and, if applicable, included in the meta-analysis). | 3, Appendix |
| Data collection process | 10 | Describe method of data extraction from reports (e.g., piloted forms, independently, in duplicate) and any processes for obtaining and confirming data from investigators. | 3, Appendix |
| Data items | 11 | List and define all variables for which data were sought (e.g., PICOS, funding sources) and any assumptions and simplifications made. | 4-5, |
| Risk of bias in individual studies | 12 | Describe methods used for assessing risk of bias of individual studies (including specification of whether this was done at the study or outcome level), and how this information is to be used in any data synthesis. | 4-5 |
| Summary measures | 13 | State the principal summary measures (e.g., risk ratio, difference in means). | 3 |
| Synthesis of results | 14 | Describe the methods of handling data and combining results of studies, if done, including measures of consistency (e.g., I^2^) for each meta-analysis. | 3-4 |

| **Section/topic** | **#** | **Checklist item** | **Reported on page #** |
| --- | --- | --- | --- |
| Risk of bias across studies | 15 | Specify any assessment of risk of bias that may affect the cumulative evidence (e.g., publication bias, selective reporting within studies). | 3-5 |
| Additional analyses | 16 | Describe methods of additional analyses (e.g., sensitivity or subgroup analyses, meta-regression), if done, indicating which were pre-specified. | 4 |
| **RESULTS** | | |  |
| Study selection | 17 | Give numbers of studies screened, assessed for eligibility, and included in the review, with reasons for exclusions at each stage, ideally with a flow diagram. | 6 |
| Study characteristics | 18 | For each study, present characteristics for which data were extracted (e.g., study size, PICOS, follow-up period) and provide the citations. | 7-8, Table 2 |
| Risk of bias within studies | 19 | Present data on risk of bias of each study and, if available, any outcome level assessment (see item 12). | N/A |
| Results of individual studies | 20 | For all outcomes considered (benefits or harms), present, for each study: (a) simple summary data for each intervention group (b) effect estimates and confidence intervals, ideally with a forest plot. | Bias in individual studies was assessed using the inclusion criteria and the covariates for study methodology in the meta-regressions |
| Synthesis of results | 21 | Present results of each meta-analysis done, including confidence intervals and measures of consistency. | 10-11 |
| Risk of bias across studies | 22 | Present results of any assessment of risk of bias across studies (see Item 15). | Appendix |
| Additional analysis | 23 | Give results of additional analyses, if done (e.g., sensitivity or subgroup analyses, meta-regression [see Item 16]). | Appendix |
| **DISCUSSION** | | |  |
| Summary of evidence | 24 | Summarize the main findings including the strength of evidence for each main outcome; consider their relevance to key groups (e.g., healthcare providers, users, and policy makers). | 11-14 |
| Limitations | 25 | Discuss limitations at study and outcome level (e.g., risk of bias), and at review-level (e.g., incomplete retrieval of identified research, reporting bias). | 13 |
| Conclusions | 26 | Provide a general interpretation of the results in the context of other evidence, and implications for future research. | 13 |

| **FUNDING** | | |  |
| --- | --- | --- | --- |
| Funding | 27 | Describe sources of funding for the systematic review and other support (e.g., supply of data); role of funders for the systematic review. | 1 |

*From:*  Moher D, Liberati A, Tetzlaff J, Altman DG, The PRISMA Group (2009). Preferred Reporting Items for Systematic Reviews and Meta-Analyses: The PRISMA Statement. PLoS Med 6(6): e1000097. doi:10.1371/journal.pmed1000097

For more information, visit: **www.prisma-statement.org**.

**MSD categories:**

MDs are categorized as per the definitions the GBD 2017 Years Lived with Disability paper.^4^ Due to the low number of eligible studies, estimates could not be made for eating disorders, conduct disorders, attention-deficit hyperactivity, and personality disorders.

**Multilevel Meta-regression Approach:**

The multi-level meta-regression approach used in this study is an extension of the standard meta-analytic approach with higher-level effects and is formally notated as follows:

$$\hat{\theta}_{ij}= \beta_{0}+ \zeta_{\left( 2 \right)ij}+ e_{ij}$$

Where $\hat{\theta}_{ij}$ is the estimator of the true effect size in the ith study and jth cluster $\beta_{0}$ is the average population effect, $\zeta_{\left( 2 \right)ij}$ represents study-level heterogeneity, and $e_{ij}$ is the sampling error. This method is discussed in more detail in Assink 2016 and Cheung 2014.^2,5^

**Distribution of Total Variance:**

Distribution of total variance was implemented in R as per demonstrated in Harrer et al., 2019.^3^

**Baujat Plot for sensitivity analyses^1^:**

Exponentiated intercept from standard meta-regression before outlier removal: 6·18 [3·83, 9·99]

Exponentiated intercept from standard meta-regression after outlier removal: 6·16 [4·21, 8·99]


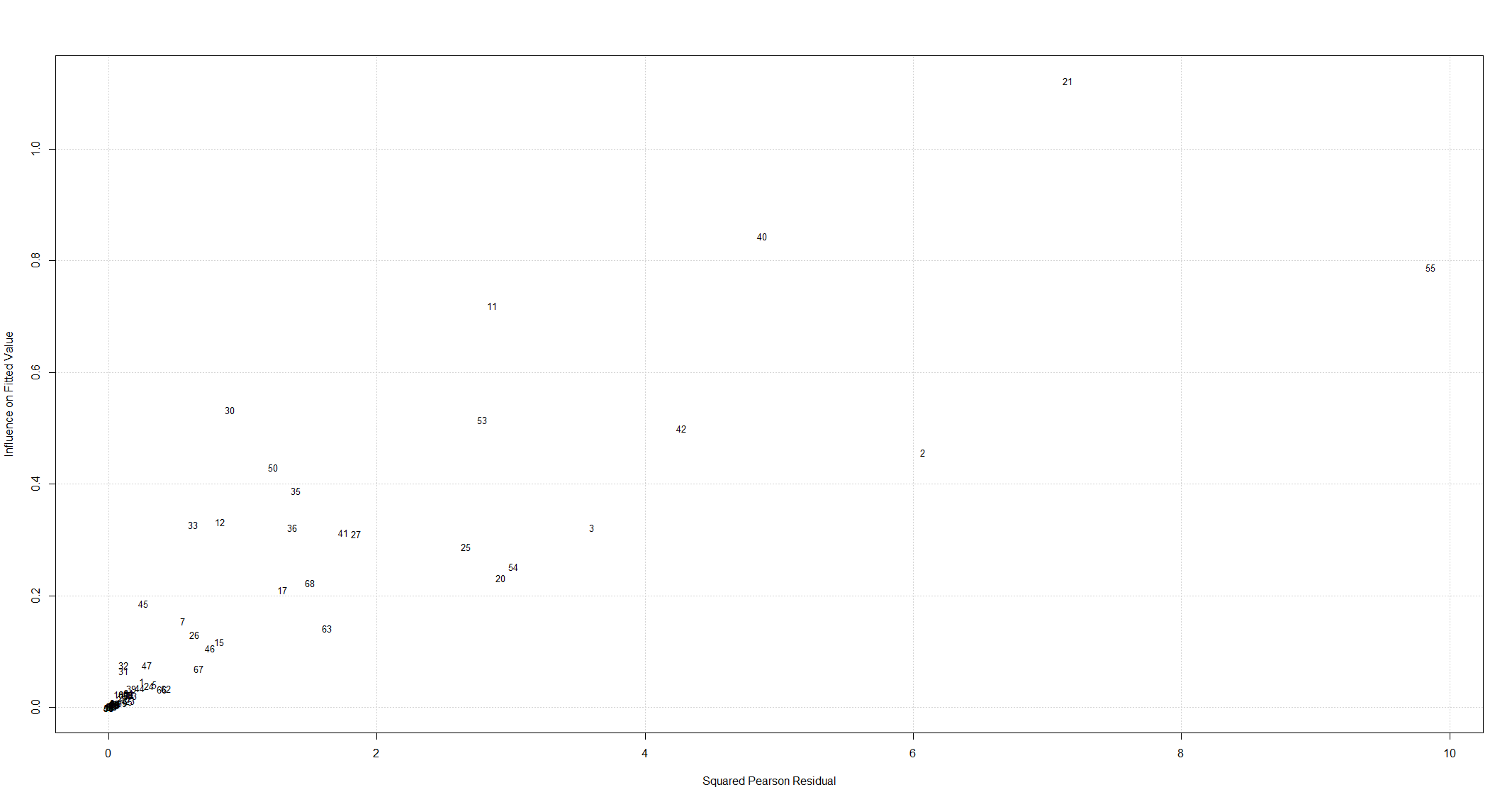


**Funnel Plot to assess risk of bias:**


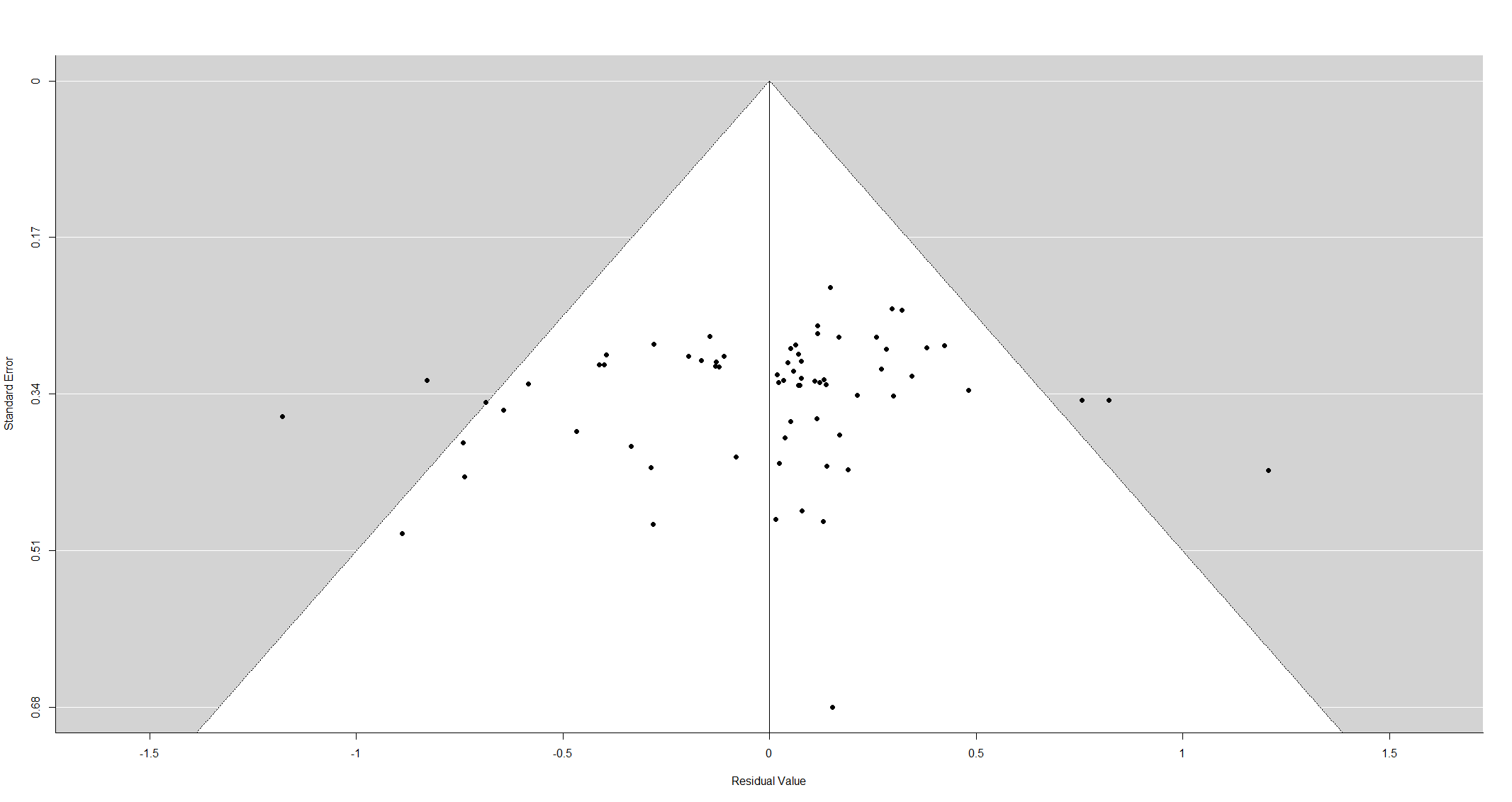


**Forest Plots by Disorder (Labeled by Author, Year, Sex, and Mid-point of age range):**


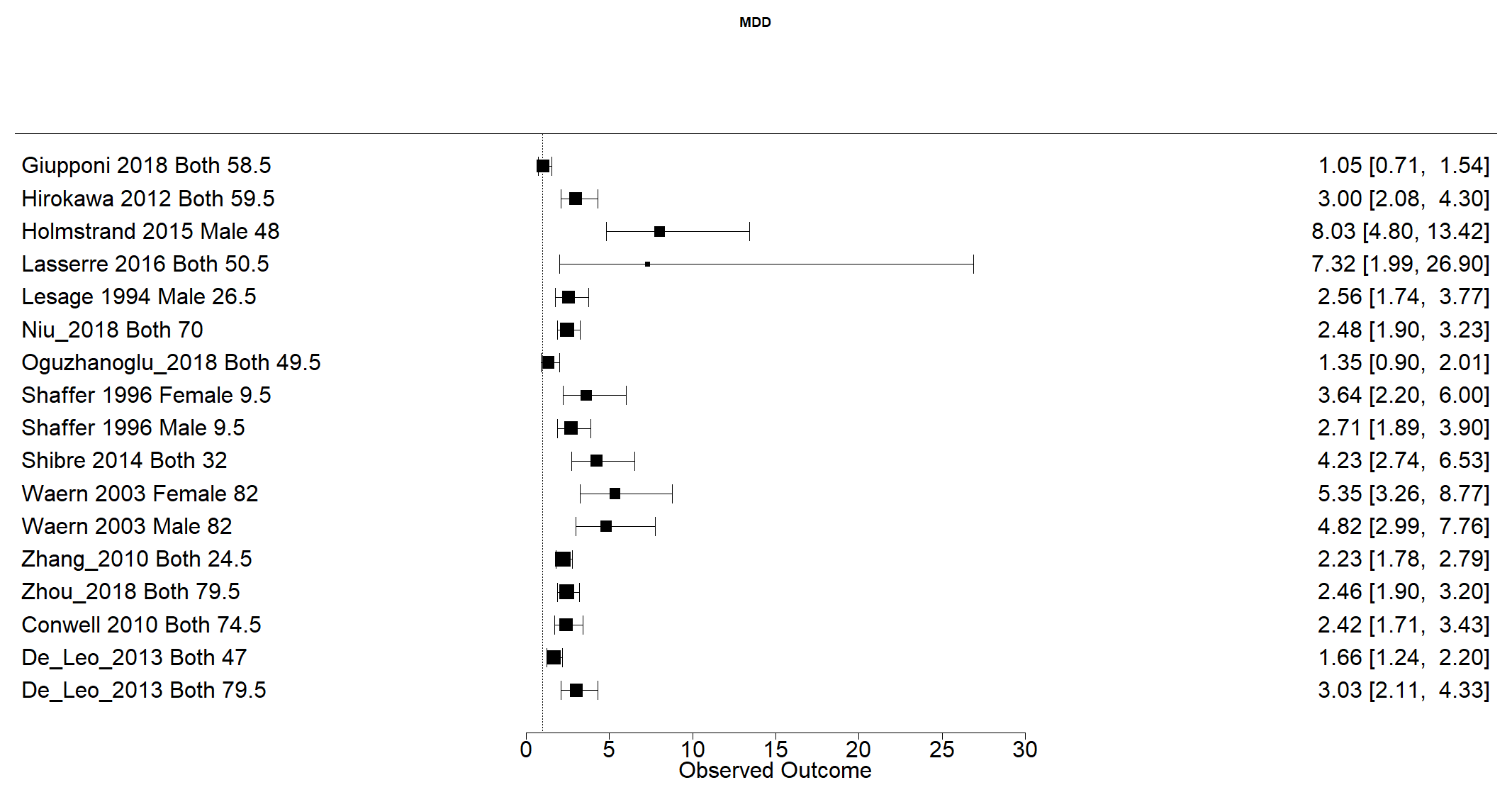


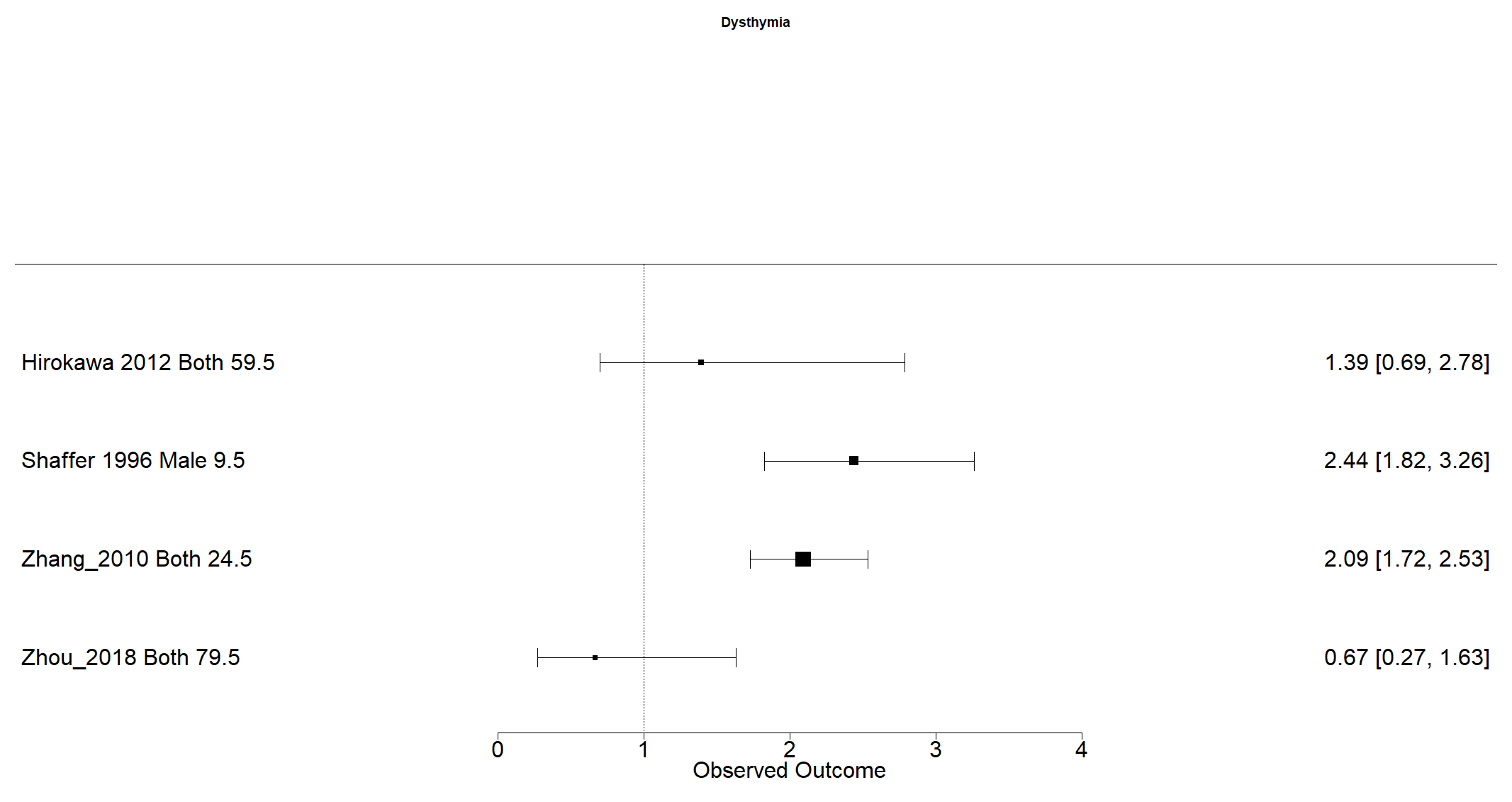


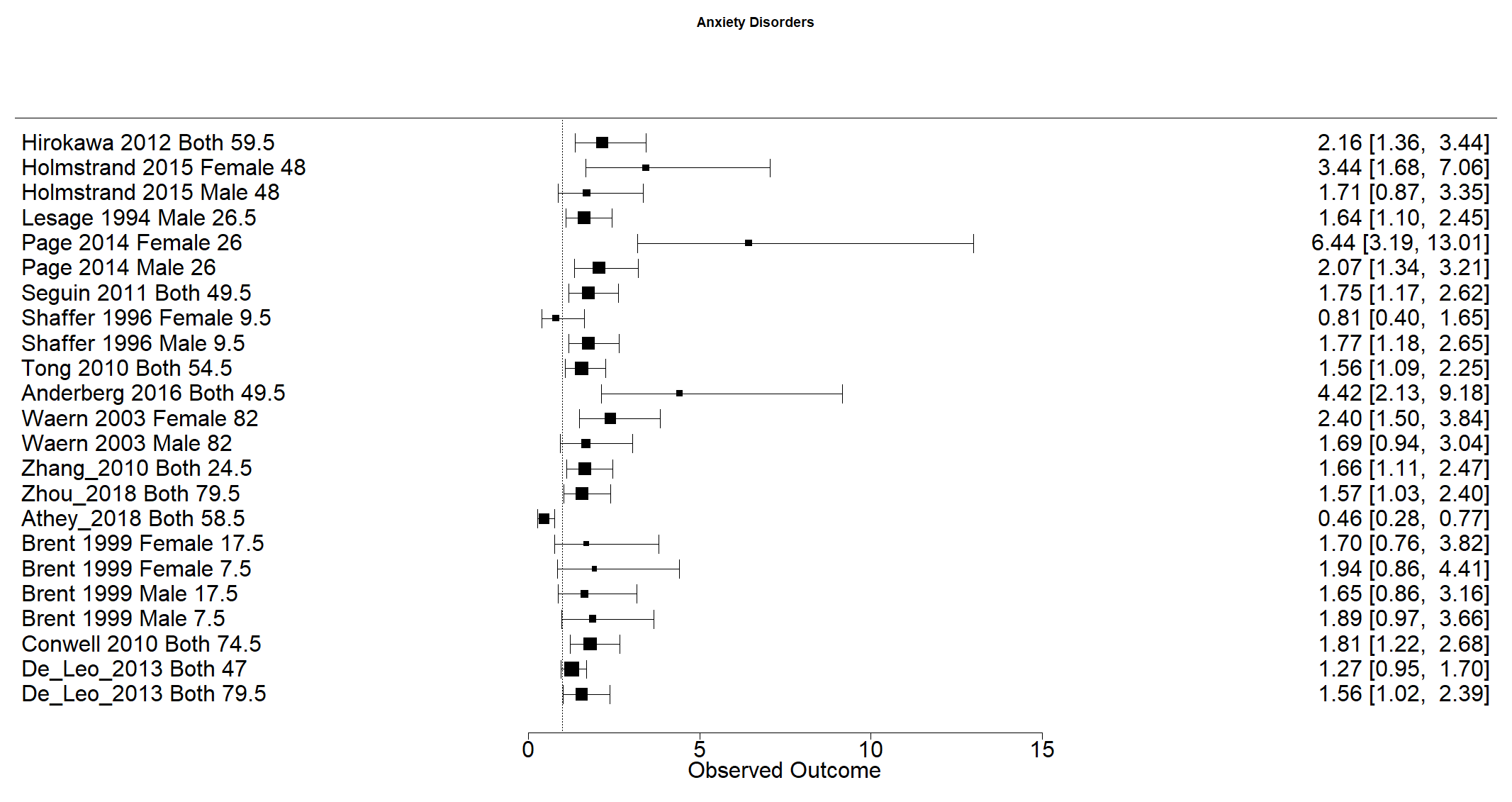


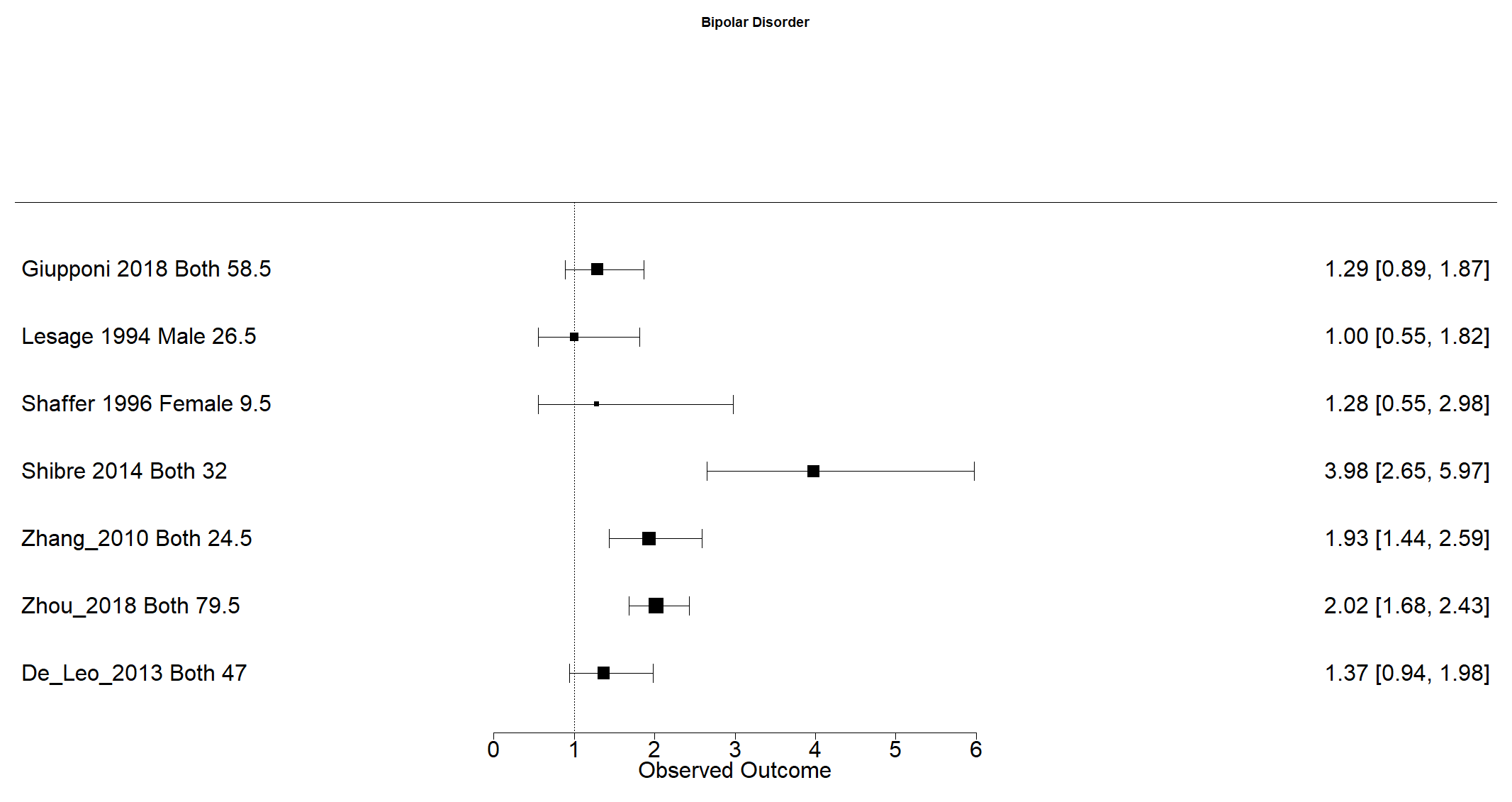


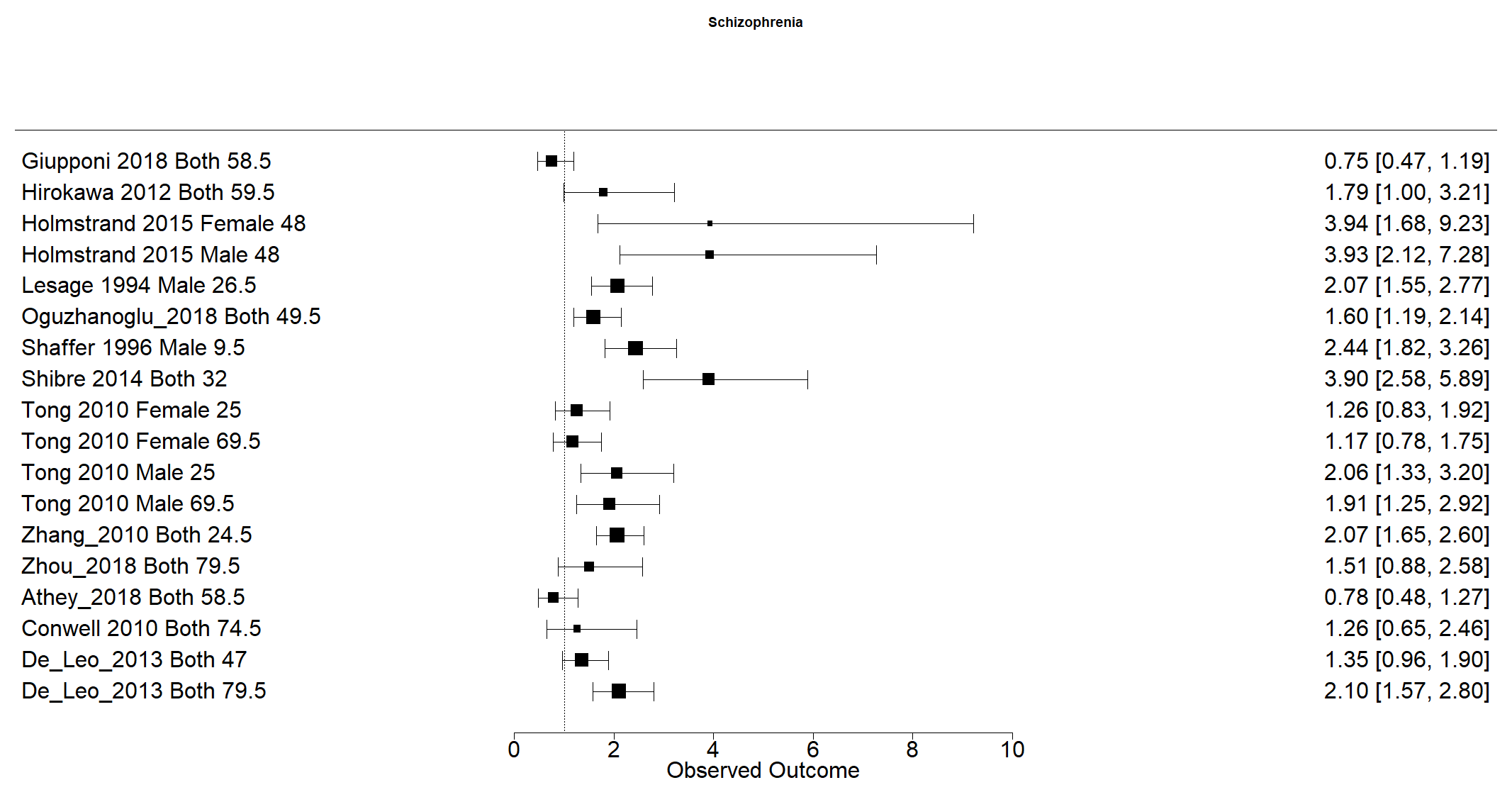


**Variance-Covariance Matrices for varying correlation thresholds:**

We tested varying correlation thresholds (*ρ = 0.25, 0.5, 0.75, 0.99*) to examine the impact on covariance in the dataset. The following tables comprise the variance covariance matrices for each of these correlation thresholds:

Correlation = 0.25

| Covariate | Intercept | Age | Percent Female | Anxiety disorders | Bipolar disorder | Dysthymia | Schizophrenia | Estimate Adjustment | Psych-Autopsy | Study design |
| --- | --- | --- | --- | --- | --- | --- | --- | --- | --- | --- |
| 1 | 0.0831694 | 0.0000482 | -0.0055802 | -0.0030207 | -0.0040220 | -0.0001996 | -0.0012211 | -0.0231218 | -0.0554192 | -0.0021308 |
| 2 | 0.0000482 | 0.0000042 | -0.0000017 | 0.0000050 | -0.0000339 | -0.0000898 | -0.0000021 | 0.0000486 | -0.0001067 | 0.0000117 |
| 3 | -0.0055802 | -0.0000017 | 0.0123723 | -0.0001103 | -0.0006613 | 0.0009476 | 0.0001629 | -0.0013755 | 0.0019352 | 0.0006285 |
| 4 | -0.0030207 | 0.0000050 | -0.0001103 | 0.0051977 | 0.0029406 | -0.0009951 | 0.0019281 | 0.0006285 | -0.0017063 | 0.0004509 |
| 5 | -0.0040220 | -0.0000339 | -0.0006613 | 0.0029406 | 0.0094631 | -0.0055033 | 0.0015205 | -0.0050962 | 0.0070746 | -0.0058256 |
| 6 | -0.0001996 | -0.0000898 | 0.0009476 | -0.0009951 | -0.0055033 | 0.0368400 | 0.0030690 | 0.0038317 | -0.0033448 | 0.0106377 |
| 7 | -0.0012211 | -0.0000021 | 0.0001629 | 0.0019281 | 0.0015205 | 0.0030690 | 0.0042281 | -0.0001160 | -0.0005314 | 0.0004919 |
| 8 | -0.0231218 | 0.0000486 | -0.0013755 | 0.0006285 | -0.0050962 | 0.0038317 | -0.0001160 | 0.0323491 | -0.0092156 | 0.0080031 |
| 9 | -0.0554192 | -0.0001067 | 0.0019352 | -0.0017063 | 0.0070746 | -0.0033448 | -0.0005314 | -0.0092156 | 0.1504533 | -0.0916210 |
| 10 | -0.0021308 | 0.0000117 | 0.0006285 | 0.0004509 | -0.0058256 | 0.0106377 | 0.0004919 | 0.0080031 | -0.0916210 | 0.0949941 |

Correlation = 0.5

| Covariate | Intercept | Age | Percent Female | Anxiety disorders | Bipolar disorder | Dysthymia | Schizophrenia | Estimate Adjustment | Psych-Autopsy | Study design |
| --- | --- | --- | --- | --- | --- | --- | --- | --- | --- | --- |
| 1 | 0.0778663 | 0.0000585 | -0.0057532 | -0.0030823 | -0.0044041 | 0.0019100 | -0.0012163 | -0.0203466 | -0.0528397 | -0.0011584 |
| 2 | 0.0000585 | 0.0000038 | -0.0000055 | 0.0000021 | -0.0000362 | -0.0000627 | 0.0000008 | 0.0000391 | -0.0001038 | 0.0000060 |
| 3 | -0.0057532 | -0.0000055 | 0.0126136 | -0.0001132 | -0.0006252 | 0.0005122 | 0.0000847 | -0.0013901 | 0.0020393 | 0.0006355 |
| 4 | -0.0030823 | 0.0000021 | -0.0001132 | 0.0045612 | 0.0029797 | -0.0005641 | 0.0019597 | 0.0003805 | -0.0008540 | 0.0001722 |
| 5 | -0.0044041 | -0.0000362 | -0.0006252 | 0.0029797 | 0.0085869 | -0.0059201 | 0.0016278 | -0.0045179 | 0.0068540 | -0.0055156 |
| 6 | 0.0019100 | -0.0000627 | 0.0005122 | -0.0005641 | -0.0059201 | 0.0384992 | 0.0026841 | 0.0030941 | -0.0049906 | 0.0110591 |
| 7 | -0.0012163 | 0.0000008 | 0.0000847 | 0.0019597 | 0.0016278 | 0.0026841 | 0.0034740 | -0.0002791 | -0.0003878 | 0.0003603 |
| 8 | -0.0203466 | 0.0000391 | -0.0013901 | 0.0003805 | -0.0045179 | 0.0030941 | -0.0002791 | 0.0287365 | -0.0081384 | 0.0069229 |
| 9 | -0.0528397 | -0.0001038 | 0.0020393 | -0.0008540 | 0.0068540 | -0.0049906 | -0.0003878 | -0.0081384 | 0.1420632 | -0.0872650 |
| 10 | -0.0011584 | 0.0000060 | 0.0006355 | 0.0001722 | -0.0055156 | 0.0110591 | 0.0003603 | 0.0069229 | -0.0872650 | 0.0902453 |

Correlation = 0.75

| Covariate | Intercept | Age | Percent Female | Anxiety disorders | Bipolar disorder | Dysthymia | Schizophrenia | Estimate Adjustment | Psych-Autopsy | Study design |
| --- | --- | --- | --- | --- | --- | --- | --- | --- | --- | --- |
| 1 | 0.075774 | 0.000080 | -0.006503 | -0.003318 | -0.004679 | 0.000647 | -0.001550 | -0.023953 | -0.046311 | -0.002013 |
| 2 | 0.000080 | 0.000004 | -0.000007 | -0.000004 | -0.000032 | 0.000017 | 0.000004 | 0.000016 | -0.000101 | 0.000003 |
| 3 | -0.006503 | -0.000007 | 0.012861 | -0.000189 | -0.000899 | -0.000074 | 0.000055 | -0.000554 | 0.001448 | 0.001356 |
| 4 | -0.003318 | -0.000004 | -0.000189 | 0.003397 | 0.003768 | -0.000644 | 0.000963 | -0.000427 | 0.001141 | -0.001020 |
| 5 | -0.004679 | -0.000032 | -0.000899 | 0.003768 | 0.009942 | -0.001148 | 0.001957 | -0.005414 | 0.007358 | -0.005998 |
| 6 | 0.000647 | 0.000017 | -0.000074 | -0.000644 | -0.001148 | 0.024031 | 0.005021 | -0.001048 | -0.000368 | -0.000059 |
| 7 | -0.001550 | 0.000004 | 0.000055 | 0.000963 | 0.001957 | 0.005021 | 0.003408 | -0.001463 | 0.001448 | -0.001260 |
| 8 | -0.023953 | 0.000016 | -0.000554 | -0.000427 | -0.005414 | -0.001048 | -0.001463 | 0.032409 | -0.007576 | 0.007746 |
| 9 | -0.046311 | -0.000101 | 0.001448 | 0.001141 | 0.007358 | -0.000368 | 0.001448 | -0.007576 | 0.122094 | -0.075109 |
| 10 | -0.002013 | 0.000003 | 0.001356 | -0.001020 | -0.005998 | -0.000059 | -0.001260 | 0.007746 | -0.075109 | 0.077945 |

Correlation = 0.99

| Covariate | Intercept | Age | Percent Female | Anxiety disorders | Bipolar disorder | Dysthymia | Schizophrenia | Estimate Adjustment | Psych-Autopsy | Study design |
| --- | --- | --- | --- | --- | --- | --- | --- | --- | --- | --- |
| 1 | 0.0784749 | 0.0001071 | -0.0055100 | -0.0012050 | -0.0060333 | 0.0096453 | 0.0003516 | -0.0200492 | -0.0556621 | 0.0060437 |
| 2 | 0.0001071 | 0.0000028 | -0.0000156 | -0.0000140 | -0.0000376 | 0.0000153 | -0.0000139 | -0.0000240 | -0.0000706 | -0.0000373 |
| 3 | -0.0055100 | -0.0000156 | 0.0128874 | -0.0000908 | -0.0003763 | 0.0002479 | 0.0000224 | -0.0020584 | 0.0024510 | 0.0004447 |
| 4 | -0.0012050 | -0.0000140 | -0.0000908 | 0.0034791 | 0.0004326 | -0.0015109 | -0.0008240 | -0.0006679 | -0.0002857 | 0.0001848 |
| 5 | -0.0060333 | -0.0000376 | -0.0003763 | 0.0004326 | 0.0095542 | -0.0051794 | 0.0021789 | -0.0050803 | 0.0106815 | -0.0091698 |
| 6 | 0.0096453 | 0.0000153 | 0.0002479 | -0.0015109 | -0.0051794 | 0.0165730 | 0.0026977 | 0.0012128 | -0.0104197 | 0.0109821 |
| 7 | 0.0003516 | -0.0000139 | 0.0000224 | -0.0008240 | 0.0021789 | 0.0026977 | 0.0018833 | -0.0012327 | 0.0011423 | -0.0003580 |
| 8 | -0.0200492 | -0.0000240 | -0.0020584 | -0.0006679 | -0.0050803 | 0.0012128 | -0.0012327 | 0.0283656 | -0.0067726 | 0.0057925 |
| 9 | -0.0556621 | -0.0000706 | 0.0024510 | -0.0002857 | 0.0106815 | -0.0104197 | 0.0011423 | -0.0067726 | 0.1953451 | -0.1457566 |
| 10 | 0.0060437 | -0.0000373 | 0.0004447 | 0.0001848 | -0.0091698 | 0.0109821 | -0.0003580 | 0.0057925 | -0.1457566 | 0.1478089 |

**References:**

1. Baujat B, Mahé C, Pignon JP, Hill C. A graphical method for exploring heterogeneity in meta‐analyses: application to a meta‐analysis of 65 trials. Statistics in medicine. 2002 Sep 30;21(18):2641-52.Cheung
2. Assink M, Wibbelink CJ. Fitting three-level meta-analytic models in R: A step-by-step tutorial. The Quantitative Methods for Psychology. 2016 Oct 1;12(3):154-74.
3. Harrer, M., Cuijpers, P., Furukawa, T.A, & Ebert, D. D. (2019). Doing Meta-Analysis in R: A Hands-on Guide. DOI: 10.5281/zenodo.2551803.
4. James SL, Abate D, Abate KH, Abay SM, Abbafati C, Abbasi N, et al. Global, regional, and national incidence, prevalence, and years lived with disability for 354 diseases and injuries for 195 countries and territories, 1990–2017: a systematic analysis for the Global Burden of Disease Study 2017. The Lancet. 2018;392(10159):1789-858.
5. Cheung MW-L. Modeling dependent effect sizes with three-level meta-analyses: a structural equation modeling approach. Psychological Methods. 2014;19(2):211.
